# Vasorelaxant and Hypotensive Effects of an Ethanolic Extract of *Eulophia macrobulbon* and Its Main Compound 1-(4′-Hydroxybenzyl)-4,8-Dimethoxyphenanthrene-2,7-Diol

**DOI:** 10.3389/fphar.2018.00484

**Published:** 2018-05-22

**Authors:** Sutthinee Wisutthathum, Krongkarn Chootip, Hélène Martin, Kornkanok Ingkaninan, Prapapan Temkitthawon, Perle Totoson, Céline Demougeot

**Affiliations:** ^1^Department of Physiology, Faculty of Medical Science, Naresuan University, Phitsanulok, Thailand; ^2^PEPITE EA4267, Université Bourgogne Franche-Comté, Besançon, France; ^3^Department of Pharmaceutical Chemistry and Pharmacognosy, Faculty of Pharmaceutical Sciences, Naresuan University, Phitsanulok, Thailand

**Keywords:** *Eulophia macrobulbon*, mesenteric artery, vasorelaxation, mechanisms, hypotensive effect

## Abstract

**Background:** Ethnopharmacological studies demonstrated the potential for *Eulophia* species to treat inflammation, cancer, and cardio-metabolic diseases. The aim of the study was to investigate the vasorelaxant effect of ethanolic *Eulophia macrobulbon* (EM) extract and its main phenanthrene on rat isolated mesenteric artery and to investigate the hypotensive effect of EM.

**Methods:** The vasorelaxant effects of EM extract or phenanthrene and the underlying mechanisms were evaluated on second-order mesenteric arteries from Sprague Dawley rats. In addition, the acute hypotensive effect was evaluated in anesthetized rats infused with cumulative concentrations of the EM extract.

**Results:** Both EM extract (10^-4^–1 mg/ml) and phenanthrene (10^-7^–10^-4^ M) relaxed endothelium-intact arteries, an effect that was partly reduced by endothelium removal (*p* < 0.001). A significant decrease in the relaxant effect of the extract and the phenanthrene was observed with L-NAME and apamin/charybdotoxin in endothelium-intact vessels, and with iberiotoxin in denuded vessels. SNP (sodium nitroprusside)-induced relaxation was significantly enhanced by EM extract and phenanthrene. By contrast, ODQ (1H-[1,2,4]oxadiazolo[4,3-a]quinoxaline-1-one), 4-aminopyridine and glibenclamide (endothelium-denuded vessels) and indomethacin (endothelium-intact vessels) had no effect. In calcium-free solution, both the EM extract and phenanthrene inhibited extracellular Ca^2+^-induced contraction in high KCl and phenylephrine (PE) pre-contracted rings. They also inhibited the intracellular Ca^2+^ release sensitive to PE. The acute infusion of EM extract (20 and 70 mg/kg) induced an immediate and transient dose-dependent hypotensive effect.

**Conclusion:** The ethanolic extract of EM tubers and its main active compound, 1-(4′-hydroxybenzyl)-4,8-dimethoxyphenanthrene-2,7-diol (phenanthrene) induced vasorelaxant effects on rat resistance vessels, through pleiotropic effects including endothelium-dependent effects (NOS activation, enhanced EDH production) and endothelium-independent effects (opening of K_Ca_ channels, inhibition of Ca^2+^ channels, inhibition of intracellular Ca^2+^ release and PDE inhibition).

## Introduction

Orchids belong to the plant family Orchidaceae, one of the most diverse group among the angiosperm with near 25,000 species (Behera et al., 2013). Aside from their ornamental value, orchids are also acknowledged for their use in traditional medicines (Bulpitt, 2005; Behera et al., 2013; Pant, 2013; Patil and Mahajan, 2013; Narkhede et al., 2016). Chinese medicine was probably the first to describe orchids for their medicinal use. Other pharmacopeias from India and many countries from South-Asia such as Taiwan, Singapore, Vietnam, Sri Lanka, Thailand, Myanmar, use orchids in traditional medicine since the ancient time (Suresh Kumar et al., 2000; Hernández-Romero et al., 2005; Luo et al., 2007). Likewise, the use of orchids in America also has a long history. Phytochemically, orchids have been reported to contain alkaloids, triterpenoids, flavonoids and stilbenoids (Kong et al., 2003; Kovács et al., 2008; Singh et al., 2012). Surprisingly, limited information on medicinal values of orchids regarding their therapeutic properties in different parts of the world is available, whereas such information would be helpful for sustainable management of resources.

Genus *Eulophia* is highly diverse in the family Orchidaceae. It has a wide distribution and comprises over 230 species, which are widespread from tropical and Southern Africa, Madagascar and from tropical and subtropical parts of Asia and Australia. Among these, one species occurs in tropical America. Different *Eulophia* species have been extensively used in the traditional system of medicines in many countries (Chinsamy et al., 2011; Patil and Mahajan, 2013; Narkhede et al., 2016). For instance, in India, the word *Amarkand* is commonly used for 30 plant species from genus *Eulophia* and for one species from the genus *Dioscorea.* Since ancient times, *Amarkand* is believed to be an excellent health-promoting agent. Rhizhomes/tubers of *Amarkand* are routinely consumed by the tribal parts of India as food as well as a therapeutic entity for better health and longevity (Narkhede et al., 2016). Phytochemical studies reported that *Eulophia* species contains phenolics, saponins, alkaloids, flavonoids, terpenoids and phenanthrene derivatives (Tuchinda et al., 1989; Maridass et al., 2008; Patil and Mahajan, 2013; Narkhede et al., 2016; Dawande and Gurav, 2017). Ethnopharmacological studies demonstrated that *Eulophia* species exhibited anti-inflammatory (Datla et al., 2010; Chinsamy et al., 2014; Schuster et al., 2017), anti-cancer (Shriram et al., 2010; Schuster et al., 2017), anti-oxidant (Tatiya et al., 2013; Schuster et al., 2017), anti-diabetic (Tatiya et al., 2013), and hypolipidemic (Tatiya et al., 2013) properties. *Eulophia macrobulbon* (E.C.Parish & Rchb.f.) Hook.f is naturally found in Thailand, Laos, Vietnam, Myanmar and Cambodia, and is traditionally used in local Thai medicine for treatment of insect bites and gangrene (Schuster et al., 2017), this latter indication suggesting a putative effect on small arteries. Recently, the ethanolic extract of *Eulophia macrobulbon* was shown to act as a potent phosphodiesterase 5 (PDE 5) inhibitor (Temkitthawon et al., 2017), an effect supported by its main compound, 1-(4′-hydroxybenzyl)-4,8-dimethoxyphenanthrene-2,7-diol [IC_50_ = 1.7 ± 0.5 μM (Temkitthawon et al., 2017)].

As PDE enzymes are widely expressed in smooth muscle cells including vascular smooth muscle cells (VSMC) (Komas et al., 1991), the aim of the present study was to investigate the vasorelaxant effect of an ethanolic extract of EM and its main constituent, 1-(4′-hydroxybenzyl)-4,8-dimethoxyphenanthrene-2,7-diol, and to unravel the mechanisms involved on isolated rat mesenteric artery. To determine whether the *in vitro* vasorelaxant properties translate into an *in vivo* effect, the acute hypotensive effect of EM extract was determined in anesthetized rats.

## Materials and Methods

### Preparation of the Plant Extract

Fresh tubers of EM were collected from Prachinburi province, Thailand. The voucher specimen (No. 002716) was identified by Associate Professor Dr. Anupan Kongbungkerd, Department of Biology, Faculty of Science, Naresuan University, and kept at Faculty of Science, Naresuan University, Phitsanulok, Thailand. As previously described (Temkitthawon et al., 2017), the tubers of EM were cut and dried at 55°C. The dried material (2 kg) was ground into a fine powder, macerated with 95% ethanol (14 L) for 3 days/time (two times). Then, it was filtered and evaporated under vacuum until dryness to give the crude ethanolic extract with the yield of 15.78% (w/w). This extract contained 0.52% (w/w) of a phenanthrene, 1-(4′-hydroxybenzyl)-4,8-dimethoxyphenanthrene-2,7-diol, which was used as a bioactive marker and a major compound of the extract (Temkitthawon et al., 2017). This compound was isolated from EM extract as described by Temkitthawon et al. (2017). Briefly, this compound was isolated and purified by using column chromatography, preparative thin layer chromatography, size exclusion chromatography, high performance liquid chromatography (HPLC) and recrystallization techniques. The EM extract and the phenanthrene were stored at -20°C until used.

### Animals

Male Sprague Dawley rats (8–12 weeks-old) were purchased from Janvier (Le Genest Saint Isle, France) for the *in vitro* study of vascular reactivity and from National Laboratory Animal Center (Mahidol University, Salaya, Thailand) for the *in vivo* study. Animals were kept under 12–12 h light: dark cycle, at 22 ± 1°C and allowed free access to standard food and water. The investigation complied with the ARRIVE animal research: reporting *in vivo* experiments. All protocols were approved by the local committees for ethics in animal experimentation No. 2015/001-CD/5PR of University of Franche-Comté (Besançon, France) and Naresuan University Animal Care and Use Committee (NUACUC, Naresuan University, Phitsanulok, Thailand, ethic approval number: NU-AE591025).

### Mesenteric Arteries Preparation

Rats were anesthetized with sodium pentobarbital (Ceva Santé Animale, France) (60 mg/kg, i.p.). Second order branches of mesenteric arteries were placed in Krebs solution with the following composition (mM): NaCl 118, KCl 4.7, KH_2_PO_4_ 1.2, MgSO_4_ 1.2, CaCl_2_ 2.5, NaHCO_3_ 25, glucose 12, maintained at pH of 7.4, 37°C, and continuously aerated with 95% O_2_, 5% CO_2_. Then 2-mm segments of artery were mounted in organ chambers and threaded on two 40-μm diameter stainless steel wires. To measure isometric force, the artery segments were connected into a Multi Myograph System (Model 610 M v.2.2, DMT A/S, Denmark). The data were recorded using Chart^TM^ Ver.7 (ADInstruments, France). After a 15-min equilibration period, segments were stretched to their optimal lumen diameter for active tension development. Optimal lumen diameter was determined based on the internal circumference/wall tension ratio of the segments by setting the internal circumference to 90% of what the vessels would have if they were exposed to a passive tension equivalent to that produced by a transmural pressure of 100 mmHg. Optimal lumen diameter was determined using specific software for normalization of resistance arteries (DMT Normalization Module; ADInstruments). After an initial equilibration period of 30 min, viability of vessels was tested from their vasoconstriction to high extracellular KCl (100 mM). The presence of functional endothelium was confirmed by more than 60% relaxation to the endothelium-dependent agonist acetylcholine (ACh 10^-5^ M) after pre-constriction with phenylephrine (PE 10^-5^ M). In some rings, endothelium was mechanically removed by gently rubbing inside the vessel with a small wire. The completeness of endothelial denudation was confirmed by the absence of relaxation to ACh (10^-5^ M). Arteries were again allowed to equilibrate 30 min before the start of the experiments.

### Experimental Protocols for Vascular Reactivity

#### Vasorelaxant Effect of EM Extract and Its Main Compound

To investigate the relaxant effect of the EM extract and 1-(4′-hydroxybenzyl)-4,8-dimethoxyphenanthrene-2,7-diol (phenanthrene), mesenteric rings were pre-contracted with submaximal concentration of PE (10^-5^ M). When the contraction reached a plateau, the EM extract (10^-4^–1 mg/ml) or phenanthrene (10^-7^–10^-4^ M) were added cumulatively to endothelium-intact rings as well as to endothelium-denuded rings to obtain a concentration-response curve. The relaxation effect was calculated as the percentage of the contraction in response to PE. The effect of the solvent, dimethyl sulfoxide (DMSO 0.23% for the EM extract, 0.18% for the phenanthrene), was evaluated in the same conditions.

#### Role of Endothelium-Dependent Pathways

To evaluate the role of endothelial nitric oxide synthase (eNOS**)**, cyclo-oxygenase (COX**)** and endothelium-derived hyperpolarizing factor (EDH) pathways in the vasorelaxant activity of the EM extract or its main compound, endothelium-intact mesenteric rings were incubated with the NOS inhibitor, N^G^-nitro-L-arginine methyl ester (L-NAME, 10^-4^ M**)**, the COX inhibitor, indomethacin (10^-5^ M**)** or with a combination of the small calcium-activated potassium channel blocker, apamin (10^-7^ M**)** and the intermediate and large conductance calcium-activated potassium channel blocker, charybdotoxin (10^-7^M) for 30 min before pre-contraction with PE (10^-5^ M**)**, respectively. Then, cumulative concentrations of the EM extract or phenanthrene were added.

#### Role of K^+^ Channels From Vascular Smooth Muscle

To investigate the contribution of K^+^ Channels to the relaxant effect of the EM extract and phenanthrene, endothelium-denuded mesenteric rings were pre-incubated with the voltage-gated potassium channel (K_V_**)** blocker, 4-aminopyridine (4-AP, 10^-3^ M), the ATP-sensitive potassium channel (K_ATP_) blocker, glibenclamide (10^-5^ M), or the large conductance Ca^2+^-activated K^+^ channels (K_Ca_**)** blocker, iberiotoxin (10^-7^ M**)**, for 30 min before pre-contraction with PE (10^-5^ M). Then, cumulative concentrations of the EM extract were added.

#### Role of sGC/cGMP Pathway

To assess whether the relaxant effect of extract and phenanthrene might be dependent on PDE inhibition, we determined if they induced changes in the soluble guanylyl cyclase (sGC)/cyclic guanosine monophosphate (cGMP) pathway in vascular smooth muscle cell (VSMC). Endothelium-denuded (E-) mesenteric rings were incubated with the vehicle (0.06% for the EM extract, 0.1% for the phenanthrene), EM extract (125 μg/ml) or phenanthrene (35 μM) for 10 min. Then rings were contracted with 10^-5^ M PE (for the EM extract) or 100 mM KCl [for the phenanthrene, as rings pre-incubated with phenanthrene did not contract to 10^-5^ M PE or low KCl (30 mM)], and then exposed to cumulative concentrations (10^-11^–10^-4^ M) of sodium nitroprusside (SNP), a NO donor. To determine whether the EM extract or phenanthrene directly activates sGC or rather acts downstream of sGC, i.e., on PDE, endothelium-denuded rings were incubated with the selective sGC inhibitor, 1H-[1,2,4]oxadiazolo[4,3-a]quinoxaline-1-one (ODQ, 10^-5^ M) for 30 min, before contraction with 10^-5^ M PE. Then, cumulative concentrations of the EM extract or phenanthrene were added.

#### Role of Calcium Channels

To assess the role of the extracellular calcium (Ca2+) influx in the extract- or phenanthrene-induced relaxations, endothelium-denuded mesenteric rings were incubated with a Ca2+-free Krebs solution containing 1 mM, methylene glycol-bis (2-aminoethylether)-N,N,N’,N’-tetraacetic acid (EGTA) for 40 min. Rings were firstly contracted with 10-5 M PE to deplete intracellular Ca2+ store from sarcoplasmic reticulum (SR) and washed four times every 10 min with the Ca2+-free Krebs solution. Then, rings were incubated with the vehicle or the EM extract at the EC_50_ (125 μg/ml) or phenanthrene at the EC_50_ (35 μM) for 10 min before 10-5 M PE or 8x10-2 M KCl was applied. Then, 10-2 M CaCl_2_ was added to evoke a contractile response (Senejoux et al., 2013).

To determine the effect of EM extract and phenanthrene on intracellular Ca2+ release, the endothelium-denuded mesenteric rings were incubated with the L-type voltage dependent Ca2+ channel inhibitor verapamil (10-7 M) for 30 min. Then, 10-5 M PE was added to the bath, and vehicle, EM extract (125 μg/ml) or phenanthrene (35 μM) were added. Under these conditions, the observed PE-induced contractions are caused by intracellular Ca2+ release via the opening of inositol 1,4,5 trisphosphate (IP_3_) receptors from SR (Tom et al., 2010).

### Acute Effect of EM Extract on Blood Pressure and Heart Rate

To investigate whether the direct *in vitro* vascular effect of EM extract on resistance vessels translates into an *in vivo* effect, we studied the acute hypotensive effect of EM extract in anesthetized rats. The effect of phenanthrene was not studied because of the low quantity and poor dissolution of the isolated compound. Normotensive male Sprague Dawley rats were anesthetized with pentobarbital (Ceva Santé Animale, France) (60 mg/kg, i.p.) and supplemented as needed to maintain deep anesthesia. The femoral vein and artery were cannulated with polyethylene tube (PE50, 0.58 mm i.d. × 0.96 mm o.d.) filled with heparinized (50 units/ml) saline. The arterial catheter was connected to a pre-calibrated pressure transducer (model BP-100 Blood Pressure Transducer, iWorx Systems, Inc., Dover, NH, United States), the output of pressure was recorded by a bridge amplifier coupled to Powerlab^®^ recording system, and an application program (ChartTM Ver.6 ADInstruments, castle Hill, NSW, Australia). After a 15-min stabilization period, systolic arterial blood pressure (SBP), diastolic blood pressure (DBP), and heart rate (HR) were recorded before and during intravenous infusion (at 1 ml/min) of 1 ml/kg of saline, vehicle (a saline solution containing 5% DMSO), EM extract (1, 5, 20, 70 mg/kg), or the NO donor SNP (25 μg/kg). The dose of SNP was chosen as a dose inducing a maximum lowering effect on blood pressure (Pagani et al., 1978; Kamkaew et al., 2013). The EM extract was dissolved in DMSO, diluted in saline and filtered through 0.2 μM syringe filter. Each subsequent infusion was administered when baseline values were fully recovered and waited for 15 min before the next infusion.

### Drugs and Solutions

PE, ACh, EGTA, L-NAME, indomethacin, apamin, 4-AP, ODQ, glibenclamide, iberiotoxin, verapamil, and SNP were all obtained from Sigma Chemical Company (St. Louis, MO, United States). Charybdotoxin was purchased from Enzo Life Sciences Company (France), DMSO was purchased from VWR International Ltd. (Prolabo Chemicals, United Kingdom). Heparin (25000 IU/mL) was purchased from Leo Pharmaceutical Products (Ballerup, Denmark). The EM extract and the phenanthrene were dissolved in DMSO and diluted with water. Glibenclamide and 4-AP were dissolved in DMSO. Indomethacin was dissolved in 0.5% w/v Na_2_CO_3_ and adjusted pH to 7.4 with 1 M NaOH. Other substances were dissolved in distilled water.

### Statistical Analysis

All data were expressed as means ± standard error of mean (SEM) of *n* animals (for *in vivo* studies) or *n* vessel segments (for *in vitro* studies). The EM extract or phenanthrene induced vasorelaxation (%) was calculated as the percentage of the contraction to PE at 10-5 M. The EC_50_ values (defined as the concentration of extract that induced 50% of the maximal relaxation) and *E*_max_ values (values of maximal relaxation) were determined by fitting the original concentration-response curves using GraphPad Prism software (version 5.0). Concentration-response relationships were analyzed using two-way ANOVA followed by the Bonferroni’s test. Unpaired Student’s *t*-test was used for two group comparisons. The multiple comparisons were analyzed using one-way ANOVA followed by Tukey’s test. *p* < 0.05 was considered statistically significant.

## Results

### Vasorelaxant Effect of the EM Extract and Its Main Compound

**FIGURE 1:**
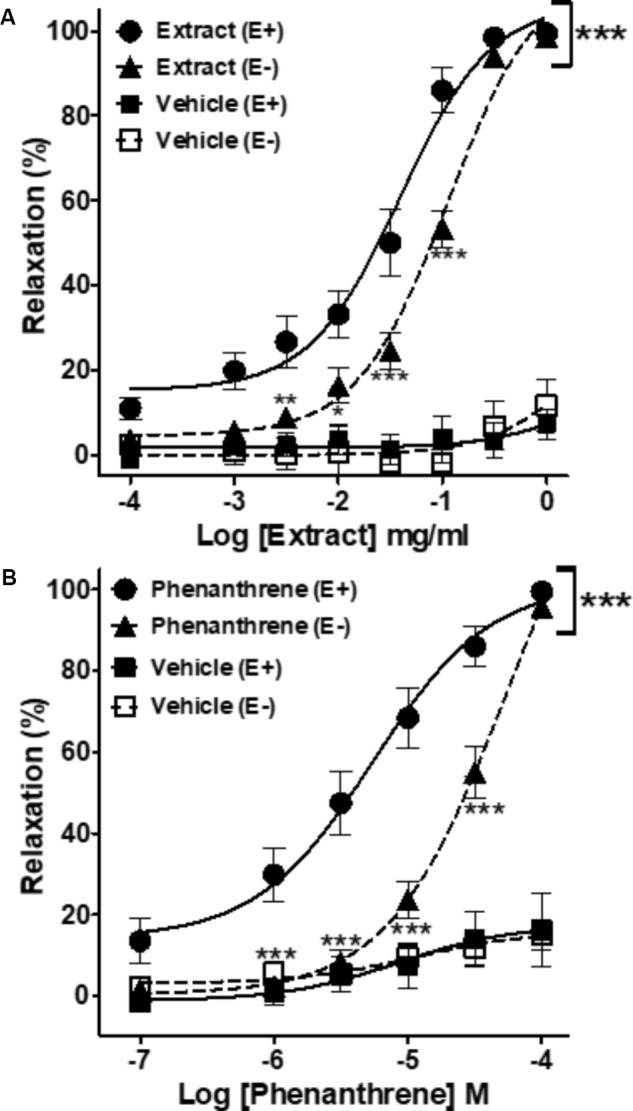
Cumulative concentration-response curves of the *Eulophia macrobulbon* (EM) extract and vehicle (DMSO 0.23%) **(A)** or of 1-(4′-hydroxybenzyl)-4,8-dimethoxyphenanthrene-2,7-diol (phenanthrene) and its vehicle (DMSO 0.18%) **(B)** in endothelium-intact (E+) and -denuded (E–) mesenteric rings. Relaxations are expressed as % contractions induced by 10^-5^ M PE. Values are means ± SEM of *n* individual arteries. ^∗^*p* < 0.05, ^∗∗^*p* < 0.01, ^∗∗∗^*p* < 0.001 vs. Extract (E+) or phenanthrene (E+) (*n* = 13–18).

As shown in **Figure 1** and **Table 1**, both EM extract (**Figure 1A**) and phenanthrene (**Figure 1B**) induced a dose-dependent vasorelaxation in endothelium-intact (E+, EC_50_ = 0.05 ± 0.01 mg/ml and 8.1 ± 2.0 μM, respectively) and endothelium-denuded rings (E-, EC_50_ = 0.12 ± 0.01 mg/ml and 35.1 ± 4.8 μM, respectively). The removal of the endothelium significantly blunted the relaxant effect of EM extract and phenanthrene (*p* < 0.01, **Figure 1**), as confirmed by the 2.3- and 4.3-fold increase in the EC_50_ values, respectively (**Table 1**). The solvent had no vasorelaxant effect (**Figure 1**). In summary, EM extract and phenanthrene induced both endothelium-dependent and-independent vasorelaxant effects.

**Table 1:** EC_50_ and *E*_max_ of *Eulophia macrobulbon* (EM) extract or 1-(4′-hydroxybenzyl)-4,8-dimethoxyphenanthrene-2,7-diol (phenanthrene)-induced relaxation in endothelium-intact (E+) and -denuded (E-) mesenteric rings in the absence or presence of various inhibitors.

	EM extract		
	EC_50_ (mg/ml)	E*_max_* (%)	*n*
Vehicle (E+)	–	7.3 ± 3.6	6
**Endothelium-intact (E+)**			
Extract (E+)	0.05 ± 0.01	99.5 ± 0.2	13
+ L-NAME	0.09 ± 0.03∗∗	99.5 ± 0.4	8
+ Indomethacin	0.03 ± 0.01	99.6 ± 0.1	8
+ Apamin + Charybdotoxin	0.10 ± 0.02∗∗∗	99.8 ± 0.3	8
Vehicle (E-)	–	11.7 ± 6.1	6
**Endothelium-denuded (E-)**
Extract (E-)	0.12 ± 0.02∗∗∗	98.70 ± 0.3	18
+ 4-AP	0.10 ± 0.02	99.1 ± 0.3	9
+ Glibenclamide	0.11 ± 0.03	97.2 ± 0.5	10
+ Iberiotoxin	0.34 ± 0.07^†††^	97.7 ± 0.7	10
+ ODQ	0.15 ± 0.05	98.3 ± 0.3	10

	**1-(4′-hydroxybenzyl)-4,8-dimethoxyphenanthrene-2,7-diol**		
	**EC_50_ (μM)**	**E_max_ (%)**	***n***

Vehicle (E+)	–	16.2 ± 8.9	6
Phenanthrene (E+)	8.10 ± 2.08	99.4 ± 0.2	13
+ L-NAME	16.17 ± 5.93∗	98.8 ± 1.0	9
+ Indomethacin	11.80 ± 6.38	98.8 ± 0.4	8
+ Apamin + Charybdotoxin	28.95 ± 8.00∗	98.5 ± 0.1	8
Vehicle (E-)	–	15.5 ± 6.9	5
**Endothelium-denuded (E-)**
Phenanthrene (E-)	35.09 ± 4.86∗∗∗	95.6 ± 1.6	14
+ 4-AP	38.04 ± 8.2	99.0 ± 1.0	11
+ Glibenclamide	48.59 ± 7.70	95.6 ± 1.6	11
+ Iberiotoxin	67.27 ± 10.05^†††^	96.2 ± 1.9	12
+ ODQ	47.20 ± 6.91	99.2 ± 0.9	10

*Values are means ± SEM of the number n of individual arteries. EC_50_ is the concentration of EM extract or phenanthrene giving half-maximal relaxation. E_max_ is the maximum response of mesenteric rings expressed as a relaxation percentage of the contraction induced by PE. ∗p < 0.05,∗∗p < 0.01,∗∗∗p < 0.001 vs. Extract (E+) or Phenanthrene (E+). ^†††^*p* < 0.001 vs. Extract (E-) or Phenanthrene (E-)*.

### Endothelium-Dependent Pathways Involved in EM Extract- and Phenanthrene-Induced Relaxation

**FIGURE 2:**
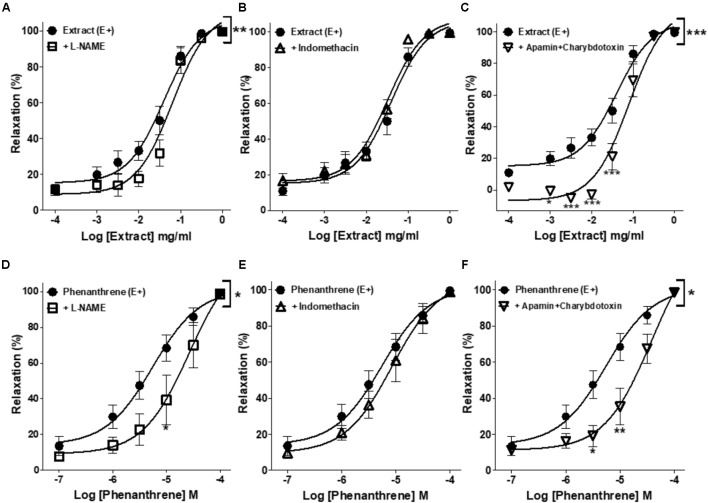
Effects of different pharmacological inhibitors on EM extract or phenanthrene-induced relaxation. Relaxant effect of EM extract or phenanthrene on endothelium-intact mesenteric rings (E+) were measured in the absence [extract (E+) or phenanthrene (E+)] or presence of 10-4 M L-NAME **(A,D)**, 10-5 M indomethacin **(B,E)** or 10-7 M apamin plus charybdotoxin **(C,F)**. Relaxations are expressed as % contractions induced by 10-5 M PE. Values are means ± SEM of *n* individual arteries. ∗*p* < 0.05, ∗∗*p* < 0.01, ∗∗∗*p* < 0.001 vs. Extract (E+) or phenanthrene (E+) (*n* = 8–13).

As shown in **Figure 2** and **Table 1**, the inhibition of NOS by L-NAME and EDH production by the combination of apamin and charybdotoxin significantly reduced the relaxation induced by EM extract (**Figures 2A,C**), and by the phenanthrene (**Figures 2D,F**), leading to an increase in the EC_50_ values of EM extract and phenanthrene without changes of the *E*_max_ values (**Table 1**). By contrast, indomethacin did not change the relaxant effect of EM extract and phenanthrene (**Figures 2B,E**).

### Involvement of K+ Channels in EM Extract- and Phenanthrene-Induced Relaxation

**FIGURE 3:**
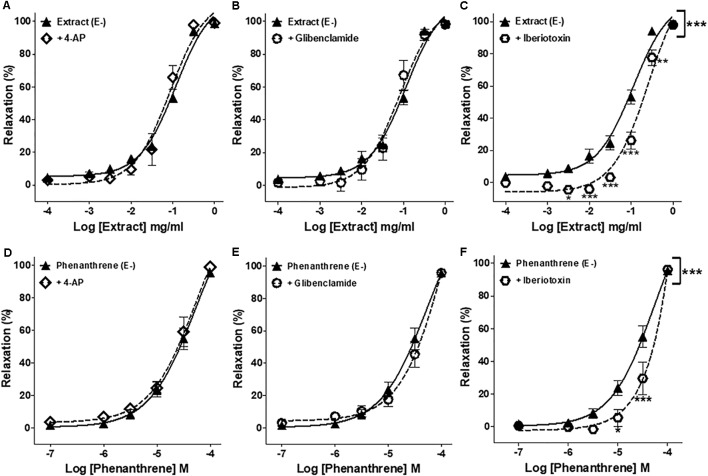
Effects of different pharmacological inhibitors on EM extract- or phenanthrene-induced relaxation. Relaxant effect of EM extract or phenanthrene on endothelium-denuded mesenteric rings (E–) were measured in the absence [extract (E–) or phenanthrene (E–)] or presence of 10-3 M 4-AP **(A,D)**, 10-5 M glibenclamide **(B,E)** or 10-7 M iberiotoxin **(C,F)**. Relaxations are expressed as % contractions induced by 10-5 M PE. Values are means ± SEM of *n* individual arteries. ∗*p* < 0.05, ∗∗*p* < 0.01, ∗∗∗*p* < 0.001 vs. Extract (E–) or phenanthrene (E–) (*n* = 9–18).

As presented in **Figure 3** and **Table 1**, iberiotoxin (a blocker of K_Ca_ channels) significantly reduced the EM extract- and phenanthrene-induced relaxation in endothelium-denuded rings (**Figures 3C,F**), while other K+ channel blockers, e.g., 4-AP (a blocker of K_V_ channels) or glibenclamide (a blocker of K_ATP_ channels) had no effect (**Figures 3A,B,D,E**).

### Involvement of sGC/cGMP Pathway in EM Extract- and Phenanthrene-Induced Relaxation

**FIGURE 4:**
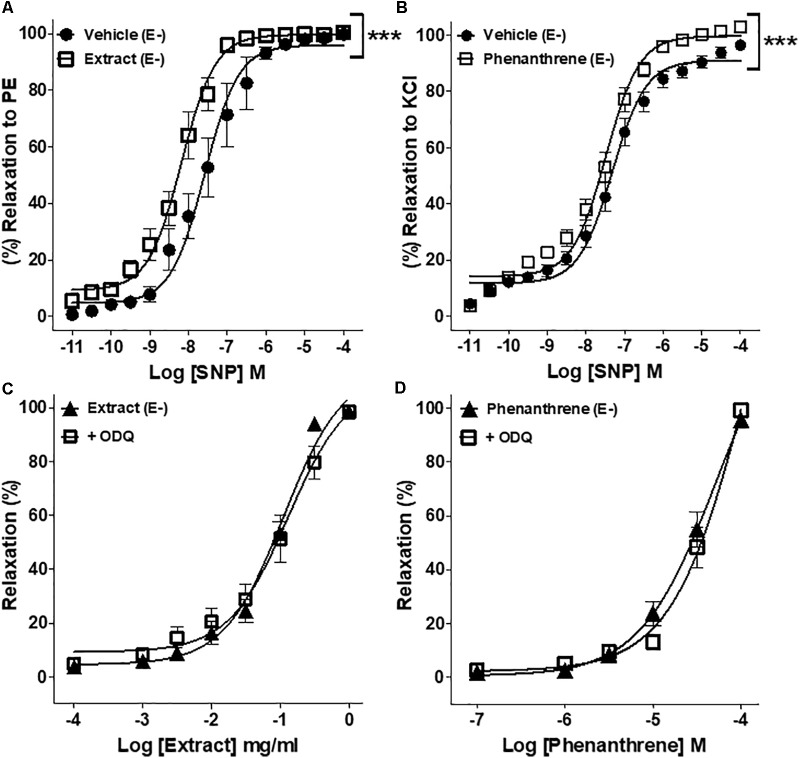
Effect of EM extract or phenanthrene on sGC/cGMP-induced relaxation. Endothelium-denuded (E-) mesenteric rings pretreated with 125 μg/ml EM or vehicle and pre-contracted with 10-5 M PE **(A)**, pretreated with 35 μM phenanthrene or vehicle and pre-contracted with 100 mM KCl **(B)** followed by relaxations with cumulative concentrations of SNP, a nitric oxide donor. EM extract **(C)** or phenanthrene **(D)**-induced relaxation of denuded (E-) rings pre-contracted with 10-5 M PE and pretreated with 10-5 M ODQ, sGC inhibitor. Values are means ± SEM of *n* individual arteries. ∗*p* < 0.05, ∗∗*p* < 0.01, ∗∗∗*p* < 0.001 vs. vehicle **(A,B)**, vs. Extract (E-) **(C)**, or vs. phenanthrene (E-) **(D)**, (*n* = 7–10).

To further understand the possible mechanisms underlying the endothelium-independent effect of EM extract and phenanthrene, the role of sGC/cGMP pathway was assessed in endothelium-denuded rings. As shown in **Figures 4A,B**, both EM extract and phenanthrene significantly increased the relaxation induced by SNP as compared to vehicle. Consistently, EC_50_ values of SNP significantly decreased from 88.7 ± 67.5 (vehicle) to 10.1 ± 3.7 nM with the EM extract (*p* < 0.001) and from 64.9 ± 21.7 (vehicle) to 20.4 ± 5.0 nM with the phenanthrene (*p* < 0.001). Likewise, the *E*_max_ values of SNP significantly increased from 99.7 ± 0.8 (vehicle) to 100.4 ± 0.4% with the EM extract and from 96.5 ± 1.8 (vehicle) to 102.9 ± 2.2% with the phenanthrene. These data indicated that EM extract and phenanthrene induced vasorelaxation through either direct sGC activation and/or downstream modulation of cGMP concentration. Thus, to determine whether EM extract and phenanthrene directly activate sGC, we studied the effect of ODQ (a sGC inhibitor). As shown in **Figures 4C,D** and **Table 1**, the sGC inhibitor did not change the relaxant effect of EM extract or phenanthrene. Altogether, these results indicated that EM extract and phenanthrene potentiated the effect of the NO donor through an effect downstream of sGC activation.

### Role of Extracellular Ca2+ Influx

**FIGURE 5:**
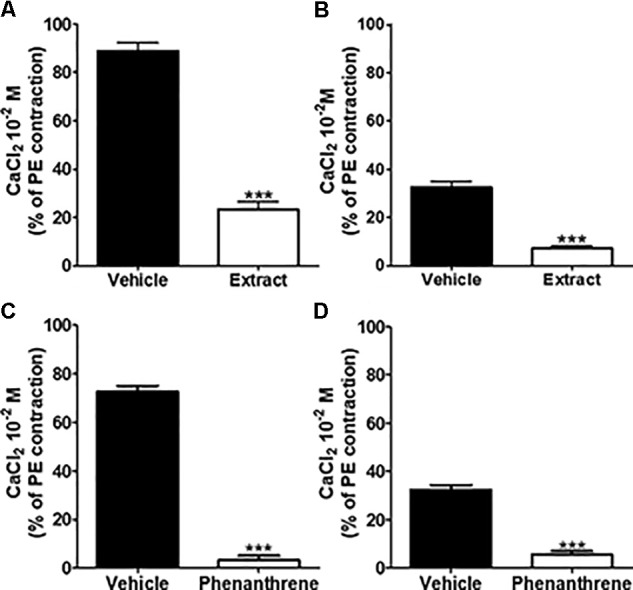
Effect of EM extract or phenanthrene on extracellular Ca2+ induced vasoconstriction. To study effect of EM extract or phenanthrene on extracellular Ca2+ influx, the endothelium-denuded mesenteric rings were incubated in Ca2+-free Krebs solution, then pre-incubated with vehicle or EM extract at 125 μg/ml or phenanthrene at 35 μM for 10 min and contracted with either 10-5 M PE **(A,C)** or 8 × 10-2 M KCl **(B,D)**, and followed by the addition of 10-2 M CaCl_2_. Changes in tension are expressed as the percentage of the contraction to 10-5 M PE. Values are means ± SEM of *n* individual arteries. ∗∗∗*p* < 0.001 vs. vehicle, (*n* = 8–14).

To investigate the role of extracellular Ca2+ influx, 10-2 M CaCl_2_ was added to mesenteric endothelium-denuded rings in Ca2+-free Krebs solution containing 10-5 M PE to activate receptor-operated Ca2+ channels (ROCCs) (**Figures 5A,C**) or 80 mM KCl to activate voltage-gated Ca2+ channels (VGCCs) (**Figures 5B,D**). As compared to vehicle, pre-incubation with either EM extract (125 μg/ml) or phenanthrene (35 μM) dramatically inhibited CaCl_2_-induced contraction in both protocols (**Figure 5**, *p* < 0.001).

### Role of Intracellular Ca2+ Release

**FIGURE 6:**
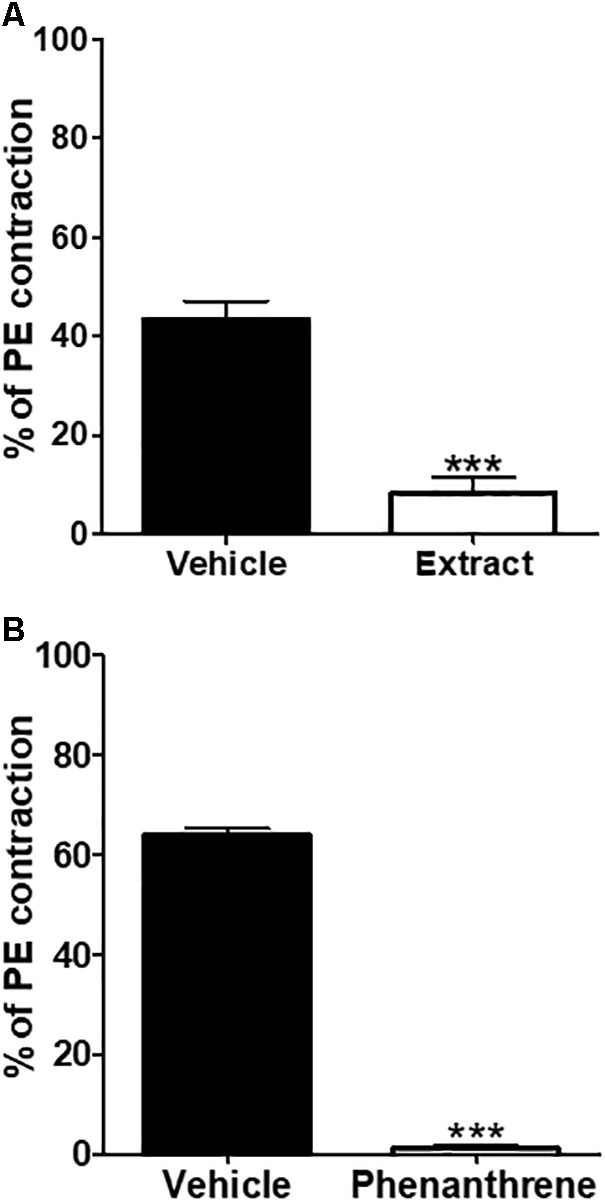
Inhibitory effect of EM extract or phenanthrene on intracellular Ca2+ release. The endothelium-denuded mesenteric rings were pre-incubated with 10-7 M verapamil for 30 min before adding 10-5 M PE in order to induce contraction in Ca2+ free solution containing the vehicle, the EM extract (125 μg/ml) **(A)** or the phenanthrene (35 μM) **(B)**. Values are means ± SEM of *n* individual arteries. ∗∗∗*p* < 0.001 vs. vehicle, (*n* = 5–8).

To determine if the EM extract- or phenanthrene-induced relaxation was related to the inhibition of intracellular Ca2+ release, endothelium-denuded rings were incubated with 10-7 M verapamil followed by the application of PE and EM extract (125 μg/ml) or phenanthrene (35 μM) or vehicle. As shown in **Figure 6**, the EM extract (**Figure 6A**) and phenanthrene (**Figure 6B**) significantly decreased the contraction to PE (*p* < 0.001).

### Acute Effect of EM Extract on Blood Pressure Levels

**FIGURE 7:**
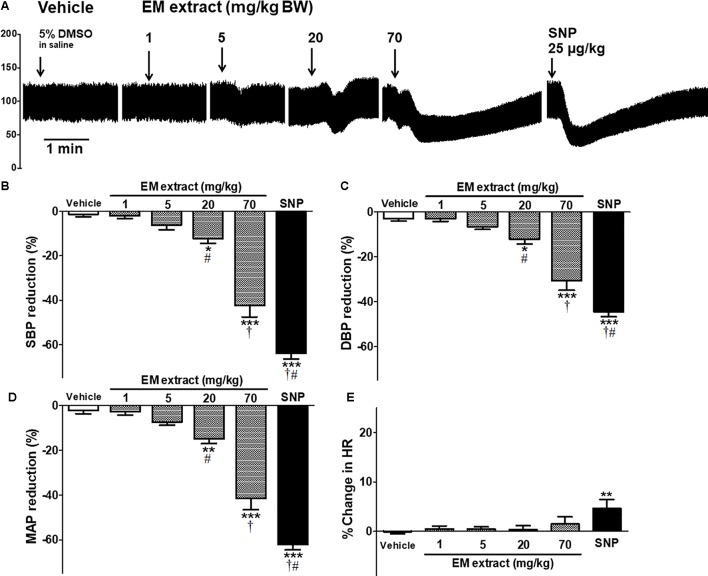
Acute effect of EM extract on blood pressure in normotensive anesthetized rats. **(A)** blood pressure traces showing the effect of single intravenous injections of vehicle (5% DMSO in saline), EM extract (1, 5, 20, 70 mg/kg BW and SNP 25 μg/kg BW, over 20 s). The bars show the percentage reduction in systolic **(B)**, diastolic **(C)**, mean arterial blood pressure **(D)** and the percent change in heart rate **(E)**, calculated from the values measured at the peak effect of DMSO, EM extract, and SNP at the doses indicated, as compared to the baseline value before each infusion. Data are expressed as means ± SEM of *n* animals. ∗*p* < 0.05, ∗∗*p* < 0.01, ∗∗∗*p* < 0.001 vs. vehicle, †*p* < 0.001 vs. EM extract 20 mg/kg, #*p* < 0.001 vs. EM extract 70 mg/kg, (*n* = 10–16).

**Figure 7A** and **Table 2** shows the effects of intravenous injection of the vehicle, EM extract and SNP on blood pressure. The values for the control (infusion of saline) were SBP = 109 ± 3 mmHg, DBP = 67 ± 2 mmHg, MAP = 81 ± 2 mmHg, HR = 296 ± 10 beats per minute (BPM). After infusion of the vehicle (DMSO 5%), the values were SBP = 109 ± 3 mmHg, DBP = 67 ± 2, MAP = 81 ± 2 mmHg, HR = 295 ± 13 BPM (no significant vs. control values). As compared to vehicle, infusion of EM extract at the doses of 20 and 70 mg/kg resulted in a significant dose-dependent reduction in SBP, DBP and MAP (**Figures 7B–D** and **Table 2**). Conversely, HR was unchanged whatever the dose of extract (**Figure 7E**). The blood pressure lowering effect of EM extract was significantly lower than that of SNP (25 μg/kg).

**Table 2:** Acute effect of *Eulophia macrobulbon* (EM) extract on blood pressure in normotensive anesthetized rats.

Group	Parameters				
	SBP (mmHg)	DBP (mmHg)	MAP (mmHg)	HR (bpm)	*n*
Control (saline)	109.3 ± 3.1	66.9 ± 2.2	81 ± 2.4	295.9 ± 10.3	21
Vehicle (DMSO 5%)	109 ± 2.9	66.8 ± 2.1	80.8 ± 2.3	295.3 ± 13.0	18
EM extract			
1 mg/kg	107.8 ± 4.9	64.4 ± 2.9	78.1 ± 3.3	270.8 ± 16.3	10
5 mg/kg	107.9 ± 3.7	63.1 ± 2.5	78 ± 2.8	295.0 ± 21.7	10
20 mg/kg	99.4 ± 3.9	55.4 ± 3.0*	70 ± 3.2	299.8 ± 20.0	13
70 mg/kg	68.2 ± 4.4***	35.8 ± 3.0***	46.6 ± 3.3***	297.3 ± 20.3	13
SNP 25 μg/kg	47.9 ± 2.2***	24.5 ± 1.9***	29.3 ± 2.4***	325.9 ± 17.1	16

*The systolic* (*SBP*), *diastolic* (*DBP*), *mean arterial blood pressure* (*MAP*), *and heart rate* (*HR*) *were measured during the peak effect of single intravenous injections of vehicle, EM extract, and SNP at the doses indicated*. *Bpm: beats per minute. All data are expressed as means ± SEM of n animals*. ∗*p < 0*.*05*,∗∗*p < 0*.*01*,∗∗∗*p < 0*.*001 vs. vehicle*.

## Discussion

The new findings of this study are that the ethanolic extract of *Eulophia macrobulbon* (EM) tubers and its main active compound, 1-(4′-hydroxybenzyl)-4,8-dimethoxyphenanthrene-2,7-diol (phenanthrene) induced a vasorelaxant effect of resistance vessels and acutely decreased blood pressure.

In the cardiovascular system, the tone of vascular smooth muscle of small arteries and arterioles determines the vascular resistance, vascular function and blood pressure (Jackson, 2000; Sandoo et al., 2010). The endothelium is an important regulator of vascular tone, through the release of vasodilatory mediators such as NO, EDH, and prostacyclin (PGI_2_) (Sandoo et al., 2010). In addition, ion channels in the plasma membrane of VSMC including different types of K+ channels and Ca2+ channels play a seminal role by determining cytosolic Ca2+ concentration and the sensitivity of contractile machinery to Ca2+ (Jackson, 2000; Webb, 2003). The results of the present study demonstrated that both the extract and the pure phenanthrene induced a relaxant effect that combined endothelium-dependent and endothelium-independent mechanisms. As regards endothelium-dependent pathways, the data showed that increased production of EDH and to a lesser extent activation of NOS is involved in the effect of EM extract, whereas both production of EDH and activation of NOS contribute to the effect of phenanthrene. By contrast, COX activation is unchanged by both extract and pure compound. This latter finding contrasts with that of Chinsamy et al. (2014) showing that a dichloromethane extract of tubers from *Eulophia hereroensis* exhibited a potent *in vitro* COX inhibitory effect. By contrast, a phenanthrene isolated from *Eulophia ochreata* (9,10-dihydro-2,5-dimethoxyphenanthrene-1,7-diol) prevented LPS-induced COX-2 expression in isolated monocytes (Datla et al., 2010). Contrary to large vessels in which NO is the major endothelium-derived factor (Wu et al., 1993), EDH is thought to play a major role in the endothelium-dependent vasorelaxation in resistance vessels (Félétou and Vanhoutte, 1999; Chauhan et al., 2003). Of interest, albeit beneficial vascular effects such as a relaxant effect on aorta (Rendón-Vallejo et al., 2012) or an inhibitory effect on expression of adhesion molecules in isolated VSMC have been reported with various phenanthrene derivatives (Huang et al., 2008; Choi et al., 2012; Lo et al., 2017), to the best of our knowledge, our study is the first to show that a benzylated phenanthrene derivative improves vascular function.

Our data revealed that the relaxant effect of EM extract and its main compound are mainly due to endothelium-independent mechanisms. The recent demonstration that EM extract as well as its main phenanthrene are potent PDE 5 inhibitors (Temkitthawon et al., 2017) led us to investigate their effect on the sGC/cGMP pathway. After NO release from endothelial cells, NO activates sGC in VSMC, resulting in the generation of cGMP. The increase in intracellular cGMP concentration activates cGMP-dependent protein kinase (PKG), which causes vasorelaxation via the modulation of Ca2+ channels as well as by decreasing the Ca2+ sensitivity of the vascular smooth muscle contractile proteins (Morgado et al., 2012). Then intracellular cGMP is rapidly inactivated to GMP by the activity of PDEs. Therefore, cGMP concentration in smooth muscle cells is mainly dependent on the balance between its production by sGC and its breakdown by PDE (Rybalkin et al., 2003; Francis et al., 2010; Morgado et al., 2012; Maurice et al., 2014). Our results showed that incubation of denuded rings with the EM extract or its main compound significantly enhanced the relaxant effect of the NO donor SNP, a direct activator of sGC, suggesting that either sGC activity or cGMP production were enhanced by *Eulophia.* The fact that ODQ, a selective sGC inhibitor, had no effect on the relaxation induced by EM extract or its main compound, discarded the first hypothesis and suggested that they act downstream by increasing the cGMP availability likely via PDE inhibition. Indeed, rat resistance arteries express four major types of PDE including PDE3 and PDE1 inhibition which hydrolyzes cGMP, PDE4 which hydrolyzes cAMP and PDE3 which mainly hydrolyzes cAMP but also cGMP (Komas et al., 1991; Sampson et al., 2001; Lugnier, 2006). Our previous *in vitro* experiments showed that EM extract/phenanthrene have a greater inhibitory effect on PDE5 as compared to PDE1 (Temkitthawon et al., 2017), suggesting that its effect on cGMP pathway is mainly due to PDE5 inhibition. However, its capacity to inhibit cAMP-dependent PDE is unknown so it cannot be excluded that its relaxant effect partly relates on PDE3 or PDE4 inhibition and the subsequent increase in cAMP-PKA signaling.

Even though further studies are required to confirm the role of PDE5 inhibition in the relaxant effect of EM extract and phenanthrene, the contribution of this mechanism seems of moderate importance as compared to other endothelium-independent mechanisms implying VSMC K+ and Ca2+ channels. Among the K+ channels inhibitors tested, only iberiotoxin significantly hampered extract- and phenanthrene-induced relaxation, indicating that they were both able to stimulate large-conductance calcium-activated K+ channels which are extensively expressed in mesenteric arteries (Beleznai et al., 2011). As these channels are activated by intracellular Ca2+ (Beleznai et al., 2011), we determined whether EM extract and phenanthrene might modulate Ca2+ homeostasis in VSMC. To assess whether EM extract and phenanthrene modified the extracellular Ca2+ influx, experiments were conducted on rings contracted with PE or KCl in Ca2+-free Krebs solution in which Ca2+ was added subsequently. The fact that the extract and phenanthrene dramatically reduced Ca2+-induced contraction after both PE and KCl exposure indicates that the blockade of both ROCCs and VGCCs is involved in the vasodilating effects. Moreover, our results showing that EM extract and phenanthrene also decreased the contraction induced by PE in the presence of verapamil, an inhibitor of VGCCs and in a lesser extent of ROCCs (Striggow and Bohnensack, 1993; Shin et al., 2005), demonstrate that they also inhibit the intracellular Ca2+ release from the IP_3_-sensitive stores of SR in mesenteric rings.

The present study was conducted on small branches of mesenteric arteries, i.e., on only one of the vascular beds contributing to the global peripheral vascular resistances. Whether the mechanisms would be the same in other arterioles requires further studies. However, the above data obtained in isolated vessels suggested that *in vivo*, EM extract would induce a hypotensive effect. Our results showed that this was the case as the cumulative infusion of the EM extract dose-dependently decreased blood pressure without changing heart rate, indicating that EM extract had a strong effect on various resistance vessels. Whether this hypotensive effect combined with the enhanced production of endothelium-derived relaxing factors observed *in vitro* would translate into positive effects on vascular diseases warrants further investigation.

## Conclusion

The ethanolic extract of *Eulophia macrobulbon* (EM) tubers and its main active compound, 1-(4′-hydroxybenzyl)-4,8-dimethoxyphenanthrene-2,7-diol (phenanthrene) induced vasorelaxant effects on resistance vessels, through pleiotropic effects including mainly endothelium-independent effects (inhibition of Ca2+ fluxes, opening of K_Ca_ channels, increase in cGMP signaling) and to a lesser extent endothelium-dependent effects (NOS activation, enhanced EDH production). The mechanisms of the extract are similar to those of the phenanthrene suggesting that this compound is mainly responsible for the vascular effects of EM extract.

## Author Contributions

SW: experimental design, *in vivo* and *in vitro* experiments, data analysis and interpretation, and manuscript drafting. KC: experimental design, data analysis and interpretation, and manuscript drafting. HM: experimental design and manuscript drafting. KI: preparation of the extract and isolated pure compound, and experimental design. PrT: preparation of the extract and isolated pure compound, and experimental design. PeT: experimental design, *in vitro* experiments, and data analysis and interpretation. CD: experimental design, data analysis and interpretation, and manuscript drafting.

## Conflict of Interest Statement

The authors declare that the research was conducted in the absence of any commercial or financial relationships that could be construed as a potential conflict of interest.
